# Gastrointestinal Nutrient Infusion Site and Eating Behavior: Evidence for A Proximal to Distal Gradient within the Small Intestine?

**DOI:** 10.3390/nu8030117

**Published:** 2016-02-26

**Authors:** Annick M. E. Alleleyn, Mark van Avesaat, Freddy J. Troost, Adrian A. M. Masclee

**Affiliations:** 1 Division of Gastroenterology and Hepatology, Department of Internal Medicine, School of Nutrition and Translational Research in Metabolism, Maastricht University Medical Center, P.O. Box 5800, 6202 AZ Maastricht, The Netherlands; a.alleleyn@maastrichtuniversity.nl (A.M.E.A.); m.vanavesaat@maastrichtuniversity.nl (M.v.A.); f.troost@maastrichtuniversity.nl (F.J.T.); 2 Top Institute of Food and Nutrition, 6700 AN Wageningen, The Netherlands

**Keywords:** intestinal brake, nutrient infusion, satiety, food intake

## Abstract

The rapidly increasing prevalence of overweight and obesity demands new strategies focusing on prevention and treatment of this significant health care problem. In the search for new and effective therapeutic modalities for overweight subjects, the gastrointestinal (GI) tract is increasingly considered as an attractive target for medical and food-based strategies. The entry of nutrients into the small intestine activates so-called intestinal “brakes”, negative feedback mechanisms that influence not only functions of more proximal parts of the GI tract but also satiety and food intake. Recent evidence suggests that all three macronutrients (protein, fat, and carbohydrates) are able to activate the intestinal brake, although to a different extent and by different mechanisms of action. This review provides a detailed overview of the current evidence for intestinal brake activation of the three macronutrients and their effects on GI function, satiety, and food intake. In addition, these effects appear to depend on region and length of infusion in the small intestine. A recommendation for a therapeutic approach is provided, based on the observed differences between intestinal brake activation.

## 1. Introduction

Obesity is considered to be a global health problem with a rapidly rising prevalence that has more than doubled over the past 30 years [1]. Obesity and its associated comorbidities result in a rapidly expanding impact on health care utilization and costs [2]. Weight loss strategies, apart from bariatric surgery, have poor success rates and an unfavorable long-term outcome. Bariatric surgery is known to be a highly effective strategy in the treatment of obesity but it is still seen as a drastic measure and only indicated in a small group of morbidly obese subjects. For example Roux-en-Y gastric bypass (RYGB), a combined procedure with a restrictive and a malabsorptive component, results in increased exposure of the distal small intestine to nutrients. The response of the gastrointestinal (GI) tract to this increased exposure of nutrients appears to play a major role in the success of bariatric surgery. In order to find new, effective, and less invasive approaches for overweight subjects, investigators are now focusing more on the GI tract as a target for medical and food-based weight loss strategies.

After ingestion of nutrients and their digestive products, several processes are activated that initiate digestion and absorption and also affect satiety and food intake. First, postprandial distension of the stomach results in the release of satiety signals; mechanoreceptors of the stomach interact with the brain by vagal and spinal sensory nerves. Thereafter, emptying of nutrients into the small intestine results in chemical interaction of the ingested nutrients with various luminal receptors in the gut wall. Part of this interaction is the activation of the so-called intestinal “brakes”, negative feedback mechanisms that influence not only motility [3,4] and secretion [5,6,7] of more proximal parts of the GI tract but also satiety and food intake.

Welch, *et al.* were the first to perform a human *in vivo* study in which a fat emulsion was directly infused into the ileum to assess the effect of this infusion on eating behavior. They observed a reduction in food intake and a delay in gastric emptying rate compared with the placebo [8]. Up to now, several others have demonstrated the inhibitory effects of ileal fat infusion on food intake, but this brake mechanism appears not to be restricted to the ileum. The existence of an intestinal brake effect on food intake is also present in the more proximal parts of the small intestine (the duodenum and jejunum). In a direct comparison between fat infusion into the duodenum and into the ileum (0.9 kcal/min), a more potent effect on appetite and fullness was observed when the same amount of fat was infused into the ileum [9]. This observation points to the existence of a proximal to distal gradient in the negative feedback of the small intestine on eating behavior.

Thus far, the “intestinal brake” has mainly been explored with ileal fat infusion as the brake substrate; however, delivery of carbohydrates (CHO) and protein in other parts of the small intestine also has the ability to induce an intestinal brake and to suppress food intake and feelings of hunger. The potency of the intestinal brake is suggested not only to be dependent on the site of intestinal delivery but also on the length of the region that is exposed and the duration and type of macronutrient infusion.

This review will focus on the effects of intestinal brake activation on GI function, satiety, and food intake. The literature regarding the intestinal brake has been reviewed before [10,11]. Our aim is to give special attention to the existence of a possible proximal to distal gradient in the magnitude of the intestinal brake effect. In addition, effects of the various macronutrients will be compared.

## 2. Site-Dependent Effects on GI Function

### 2.1. Motility

The theory of intestinal brake activation originates from the hypothesis that steatorrhea was associated with small bowel hypomotility; malabsorbed fat that reached the ileum and colon is associated with inhibition of duodenal and jejunal motility [12]. The presence of nutrients in the small intestine is essential for subsequent absorption and digestion, and this control is accomplished via adaptation of GI transport, including gastric emptying and intestinal transit.

### 2.2. Small Intestinal Transit Time

Delayed small intestinal transit time is best described in the context of the ileal brake. Spiller, *et al.* infused 10 g fat (90 kcal in 30 min) in the ileum and observed a delay in transit time with an inhibition of jejunal pressure waves [13]. This is in line with a study conducted by the same research group a few years later in which they compared free fatty acid infusion into the ileum *vs.* jejunum. Taken together, these observations indicate that infusion of fat into the ileum exerts an inhibitory effect on jejunal motility. However, when fat was infused directly into the jejunum, transit time was accelerated and the jejunal flow was increased [14]. These results suggest that important differences exist between jejunal and ileal activation and their effects on small intestinal transit time and motility.

### 2.3. Gastric Emptying Rate

A delay in gastric emptying, in the absence of mechanical obstruction, is thought to result from changes in gastric motor functions: via an increase in phasic and tonic pyloric pressure waves [15], or via suppression of antral pressure waves [15] and a reduction in fundic tone [16]. Cook, *et al.* observed an increase in the frequency and amplitude of the isolated pyloric pressure waves (IPPW) after infusion of fat into the duodenum [17]. The delivery of nutrients into the duodenum delays gastric emptying and increases phasic and tonic pressure in regions located near the pylorus. Lin, *et al.* infused oleate (fat) in the small intestine of dogs with a proximal jejunal fistula (≈60 cm from the pylorus) or with a distal midgut fistula (≈150 cm from the pylorus). They observed an inhibition of gastric emptying in a dose-related fashion. Herewith, the existence of a similar “brake” mechanism in the duodenum and jejunum was shown [18]. Lin, *et al.* also demonstrated a full inhibitory response on gastric emptying when oleate was infused over a total length of 150 cm in the small intestine. This result suggests that increasing the exposure of nutrients to regions of the small intestine strengthens the inhibitory response on gastric emptying.

Only a few studies explored the inhibitory effect of nutrient infusion in different regions of the small intestine on GI motility. For instance, gastric emptying of a liquid meal was significantly more delayed after ileal fat infusion compared to infusion of the same amount of fat in the duodenum [19]. Infusion of a mixture of glucose and rice starch into the ileum induced changes in upper GI function and delayed gastric emptying [20]. With respect to protein, the effects on gastric emptying are not uniform. In dogs, amino acid infusion of 0.3, 0.6, or 0.9 kJ/min generated a dose-dependent delay in gastric emptying [21]. Welch, *et al.* infused a protein hydrolysate (8%) into the human ileum and found no inhibitory effect on gastric emptying [22].

Taken together, the findings from these studies indicate that nutrients in the small intestine regulate the gastric emptying process [15], and the extent of this nutrient-mediated feedback is not only dependent on the region exposed to nutrients but also on the length of the small intestine exposed to nutrients [18] and type of macronutrients infused.

## 3. Site-Dependent Effects on Food Intake and Satiety: Duodenal Brake

Most of the studies evaluating the effects of intestinal nutrient infusion on food intake and satiety have been conducted by duodenal delivery (Table 1). This is probably due to the fact that positioning an intestinal catheter in the duodenum is easier and more convenient than positioning the catheter in the jejunum or ileum. Alternatively, to isolate the observed small intestinal effects from oral and particularly intragastric effects, it is best to infuse the nutrients as proximal as possible in the gut, that is, directly after passing the pylorus, avoiding the effects of gastric emptying. The existence of a duodenal brake for CHO originates from the idea that increased interactions of glucose with glucose-sensing receptors will also increase the satiating effects of CHO [23]. This idea was based on two principles; (1) glucose in the small intestine was shown to stimulate vagal activity; and (2) infusion of glucose elevated plasma insulin in cats [24]. From then on, several studies more systematically evaluated the effects of CHO infusion into the duodenum on satiety and food intake. Lavin, *et al.* observed a significant reduction in energy intake (17%), suppression of hunger, and increase in satiety when glucose was infused into the duodenum compared to intravenous infusion of glucose in a placebo-controlled study, while blood glucose concentrations did not differ between the two interventions [23]. Two years later, the same researchers observed comparable results after intraduodenal infusion of glucose (3.2 kcal/min): a significant reduction in energy intake of 26% compared to control [25]. When comparing the studies that infused CHO into the duodenum, Macintosh, *et al.* found a significant effect on food intake, with a threshold of ≥2.86 kcal/min [26]. Furthermore, an inhibitory effect on the desire to eat was already observed at a caloric load of 2.0 kcal/min [27]. Taken together, these observations indicate that activation of the duodenal brake by CHO leads to an increased perception of fullness, suppression of hunger, an increase in satiety, and a reduction in energy intake (Table 1), unaffected by fluctuations in blood glucose concentrations. Changes in blood glucose concentrations have a significant impact on the gastric emptying rate in healthy adults and thereby interfere with the observed effects on food intake and satiety. There is evidence that hypoglycemia accelerates gastric emptying and consequently hastens the absorption of CHO from the GI tract. Hyperglycemia potentiates a delay in gastric emptying.

Protein is considered to be the most anorectic of the three macronutrients [45,46]. Consequently, several investigators have studied the effects of intraduodenal protein infusion on food intake and satiety. Furthermore, it has been established that intraduodenal infusion of pea protein results in a greater decrease in energy intake when compared with oral ingestion of the same amount of pea protein [28,47]. Ryan, *et al.* conducted a study to evaluate the effect of intraduodenal whey protein infusion on satiety and energy intake. Assuming that intraduodenal fat and CHO suppress appetite and energy intake in a dose-dependent manner, this group aimed to study whether a dose-dependent effect of duodenal protein delivery exists on appetite. Caloric infusion rates of 0.5, 1.5, and 3.0 kcal/min of protein showed that the effects of intraduodenal protein are indeed dose dependent and resulted in suppression of food intake (Table 1). However, this effect was only found to be significant *vs.* placebo after an infusion of 3 kcal/min (26.3% reduction in food intake) [28].

Remarkably, no significant decrease in hunger or appetite scores during and after protein infusion was observed (Table 1). Intraduodenal nutrients can potently suppress food intake, while little to no effect is seen on appetite perceptions. In addition, Steinert, *et al.* observed a more potent appetite-suppressing effect after intragastric infusion of glucose than with intraduodenal infusion. These results indicate that the stomach has an important contribution in inducing satiety mechanisms and that this is possibly mediated by mechanical factors, such as gastric distension [48]. This effect may be macronutrient dependent, related to the influence of the macronutrient on the gastric emptying rate. Another explanation for the lack of effect on satiety and hunger scores may be related to the differences in appetite-suppressive effects within different sources of protein [49]. In addition, the absence of the satiating effects of intraduodenal protein may have been caused by differences in study design—differences in feeding status before the start of infusion, duration of fasting, and total time of infusion.

Comparing intraduodenal fat and glucose, fat is shown to be a more potent inhibitor of food intake compared to isocaloric CHO infusion (Table 2) [17,26,50]. Protein and fat both activate the duodenal brake in a dose-dependent fashion, probably due to interaction with a larger number of receptors within the small intestine [29,35]. A specific threshold in caloric load is necessary for macronutrients to activate the duodenal brake. For protein, a caloric load of 3 kcal/min significantly reduced energy intake (26.3% reduction) [28]. CHO reduced energy intake by 17% when a caloric load of 3.2 kcal/min was infused [25]. Remarkably, infusion of fat at a caloric load of 2.0 kcal/min already inhibits energy intake at 31% compared to the control (Table 1) [37].

Combining fat with protein or glucose shows no effect on food intake and satiety [30,38]. The absence of these additive effects can be caused by the assumption that not a specific caloric load, but a specific concentration threshold of fatty acids, monosaccharides, or free amino acids is required to be present in the lumen of the small intestine in order to activate a negative feedback mechanism. For example, infusion of the amino acid l-tryptophan at a caloric load of 0.15 kcal/min shows a significant effect on satiety and food intake (Table 1) (19% reduction) [31]. In addition, when a fatty acid (lauric acid, C12) is directly infused into the duodenum with a caloric load of 0.4 kcal/min, a significant effect on food intake was observed (reduction of 20%) while no effects on satiety scores were seen (Table 1) [40]. These results suggest that increasing the release of degradation products of fat, protein, and CHO strengthens the effects on the inhibition of food intake. However, it should be noted that this effect is also dependent on the type of fatty acid or amino acid infused. It is hypothesized that, when a combination of macronutrients is infused at the same caloric load as a single macronutrient, the critical threshold of activating the duodenal brake is not reached by the reduced presence of degradation products of fat, glucose, and protein.

The abovementioned results suggest that all three macronutrients activate the duodenal brake, though to a different extent. The three different macronutrients exert an effect on food intake and satiety by mediating different mechanisms of action. The data reported here support the assumption that fat requires the lowest amount of calories infused per minute to trigger a significant reduction in food intake. Furthermore, combining fat with glucose or protein showed no additive effects. Infusion of nutrients at a higher rate than the normal gastric emptying rate (≈2–3 kcal/min) or in supraphysiological concentrations could activate the more distal regions of the small intestine, and thereby have a more potent effect on food intake and satiety. In addition, when directly infusing degradation products of fat, protein, or CHO, supraphysiological concentrations are reached within a short period. Thereby, the physiological concentration of these degradation products in the distal regions of the small intestine is varying and not completely known. Consequently, is it difficult to distinguish between specific effects contributing to the duodenal brake and the more distal parts of the small intestine.

## 4. Site-Dependent Effects on Food Intake and Satiety: Jejunal Brake

Several studies have shown that infusion of nutrients into the jejunum modulates intestinal transit time. At present, only few studies have explored the effects of jejunal nutrient infusion on food intake, appetite, and feelings of satiety. Welch, *et al.* infused a fat emulsion with a caloric delivery rate of 4.9 kcal/min in the ileum and jejunum in humans and found early satiety and a reduction in food intake at both sites (Table 1) [39]. However, the reduction in food intake was larger after jejunal infusion (50% compared to the control). It is worth noting that the infusion rate of 4.9 kcal/min can be seen as supraphysiological and exceeds the rate of nutrients entering the small intestine in the postprandial state [51]; therefore, fat could have spilled into the more distal small intestine, thereby activating the more distal ileal brake.

To the best of our knowledge, no studies have been published in which the effects of jejunal protein infusion on food intake and satiety have been investigated. Regarding CHO, one study compared the effects of jejunal and duodenal infusion on food intake and satiety. A significant decrease in satiety and food intake (11%) in favor of jejunal infusion was shown compared to duodenal infusion. These results suggest that jejunal infusion of nutrients has an effect on food intake, but expanding the area exposed to nutrients increases the impact on food intake. Comparable effects are observed after Roux-and-Y Gastric Bypass, in which nutrients bypass the duodenum and a large part of the jejunum. Hereby, a supraphysiological amount of non-digested nutrients is delivered to the distal small intestine and contributes to a more chronic and sustained activation of the jejunal and ileal brake. Compared to the duodenum and ileum, reports on effects of jejunal perfusion with nutrients on food intake and satiety are scarce (Table 1). As stated before, positioning of a catheter or tube in the more distal regions of the small intestine is invasive and time consuming.

## 5. Site-Dependent Effects on Food Intake and Satiety: Ileal Brake

Several studies have shown that infusion of fat into the ileum induces a brake effect on satiety and food intake [8,39], delays gastric emptying, and reduces the secretion of gastric acid [7] and pancreatic enzymes [5]. Welch, *et al.* were the first to demonstrate the effects of nutrient-driven satiety in the ileum. These investigators infused an emulsion of 50% corn oil and 3% albumen into the ileum of healthy volunteers and measured the effect on food intake and satiety. Subjects had a significant reduction in energy intake (30%) during ileal infusion of fat, with a caloric load of 4.93 kcal/min compared with the control (Table 1) [8]. This caloric infusion rate is considered supraphysiological and it should be taken into account that some of the fat will have spilled over into the colon. The colon is capable of secreting Peptide YY (PYY) and Glucagon-like peptide-1 (GLP-1) [52,53] and may also have a role in regulating appetite and food intake.

The ileal brake concept has traditionally been studied by directly infusing nutrients into the ileum, but information on this negative feedback mechanism on food intake and satiety is provided by studying surgical interventions in which the distal small intestine is exposed to an increased amount of nutrients. One such example is “ileal transposition”. In animal experiments, transposing 10 or 20 cm of lower ileum to mid duodenum or a jejuno-ileal bypass showed a sustained post-operative reduction in food intake and change in body weight [54]. In addition, ileal transposition in Zucker obese rats, by transposing a 10 cm segment of the terminal ileum to the upper jejunum, caused long-term reduction in body weight and a persistent decrease in preference for dietary fat [55]. In humans, the jejuno-ileal bypass (JIB) by Payne and De Wind was the first surgical treatment for morbid obesity [56,57]. The weight loss observed after this procedure has at first been contributed to chronic malabsorption of nutrients. However, the loss in body weight was larger than could be accounted for via caloric loss in the stool. Näslund, *et al.* found significantly higher serum concentrations of distal gut hormones PYY and GLP-1 after JIB compared to pre-surgical levels, providing a possible explanation for the excess weight loss via neuro-hormonal feedback [41]. However, due to the high rate of complications after JIB, this surgical procedure is currently no longer performed in bariatric practice [58].

It has been well established that a high amount of fat activates the ileal brake. However, more recent studies indicate that much smaller amounts of fat can also activate the ileal brake. Maljaars, *et al.* tested this hypothesis by conducting several studies on the satiating effects of small amounts of fat, and these authors investigated whether this effect is dose dependent. Healthy volunteers were intubated with a naso-ileal catheter and were randomized to the ileal infusion of 3 g or 9 g fat over a 45-min period, resulting in an infusion rate of 0.6 kcal/min and 1.8 kcal/min. The control group received a fat-containing breakfast of 3 g with an ileal placebo infusion. Both the low and high ileal fat infusion resulted in a reduction in appetite and an increase in satiety, without any evidence for dose dependency [43], in contrast to the dose-dependent effect of intraduodenal fat administration [35]. A possible explanation for the absence of this dose-dependent effect could be related to the variety of lipolytic capacity of the different regions of the small intestine. It is known that the lipolytic capacity in the ileum is much smaller compared with the duodenum [59], and therefore it is possible that a high dose of fat could not be fully hydrolyzed to fatty acids and monoglycerides before entering the colon. Currently, digestion of triacylglycerol is considered a necessary step for fat to establish its satiety-increasing properties [60].

Studies in humans evaluating the effects of all three macronutrients and their potency in inducing an ileal brake are limited. Van Avesaat, *et al.* tested the macronutrient-specific effects in the ileum on eating behavior. In this single-blind randomized placebo controlled study, they compared the effects of six different interventions: saline, fat emulsion (51.7 kcal), low-dose protein (17.2 kcal), high-dose protein (51.7 kcal), low-dose CHO (17.2 kcal), and high-dose CHO (51.7 kcal) for 90 min (Table 1). The low doses of macronutrients were infused at a rate of 0.19 kcal/min and the high doses at a rate of 0.57 kcal/min. Notably, these caloric infusion rates do not exceed the physiological gastric emptying rate, which is known to vary between 1 and 4 kcal/min in healthy adults. These authors were the first to observe a suppression of food intake after CHO and protein infusion into the human ileum to the same extent as an equicaloric amount of fat (infusion rate 0.57 kcal/min). No effects were observed after infusion of 0.19 kcal/min of CHO or protein. Protein was the only macronutrient to influence satiety scores [42]. This effect is not unexpected; protein is considered to be the most anorectic macronutrient. However, no significant differences in food intake between protein, CHO, and fat were observed at a caloric infusion rate of 0.57 kcal/min [42]. Therefore, it is still unclear whether differences between these macronutrients in terms of their ileal brake inhibitory effect on food intake exist. These results suggest that equicaloric amounts of protein and CHO in the ileum can induce an ileal brake and affect eating behavior to the same extent. This threshold for all three macronutrients is considerably lower than that in the duodenum.

## 6. The Effects of Length and Site of Intestinal Contact on Nutrient-Driven Satiety and Food Intake

**Figure 1:**
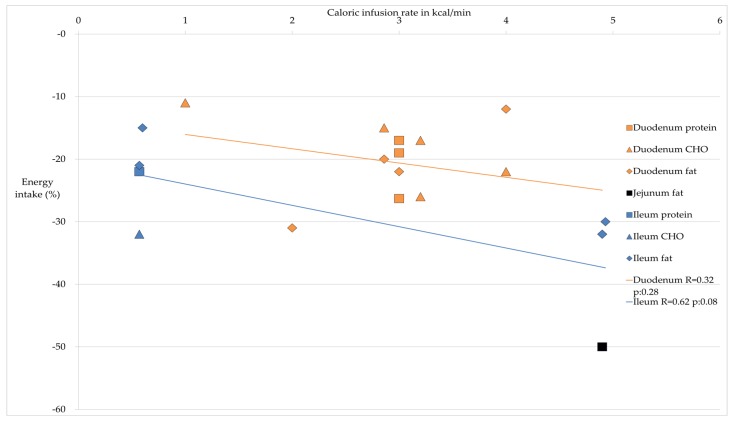
Caloric infusion rates (kcal/min) in the different regions of the small intestine and the decrease in food intake in percentage and linear regression for the duodenum and ileum.

The different sites of the small intestine where nutrients are delivered are of utmost importance for the satiating effects. Table 2 provides an overview of the effects on food intake generated by the different macronutrients in the regions of the small intestine. This table provides evidence for a proximal to distal gradient in the small intestine; infusing nutrients in the distal small intestine generates a more potent effect on food intake. Figure 1 expands on the data from Table 2, revealing the percentage of decrease in energy intake in relation to the caloric infusion rate given for different locations with different macronutrients as well as the linear regression between the variables caloric infusion rate in kcal and the decrease in energy intake in % per location. With duodenal delivery of nutrients, the higher the infusion rate in calories, the stronger the inhibitory effect on food intake. With ileal delivery of nutrients, the inhibitory effect on food intake is more pronounced compared to duodenal delivery, both at lower and at higher caloric delivery rates.

Based on the studies mentioned above, we may conclude that each site of the small intestine is, when exposed to undigested macronutrients, able to induce a so-called intestinal brake, while the magnitude of these effects is site dependent. Meyer, *et al.* and Lin, *et al.* demonstrated that increasing the small intestinal area exposed to nutrients induces more potent effects on gastric emptying rate and food intake [18,61]. Increasing the small intestinal area that is exposed to undigested nutrients may also cause a more potent effect on food intake in humans. Maljaars, *et al.* investigated this hypothesis and found no significant differences in reduction of food intake when 2 g of fat (0.6 kcal/min) was infused simultaneously (2 g each to all sites in the same time interval) or sequentially (2 g each to all sites in sequential time intervals) at different sites in the small intestine [44]. A possible explanation is that in the study of Meyer, *et al.* a higher rate of infusion was chosen [61]. Taken together, it can be suggested based on surgical techniques and intestinal infusion studies that increasing the caloric load of an infusion expands the small intestinal area exposed to nutrients and increases the effect on food intake and GI motility.

## 7. The Effect of BMI, Age, and Gender on the Intestinal Brake Effect of Nutrients

BMI, age, and gender may influence the magnitude of the intestinal brake effects after infusion of nutrients. For BMI, Chapman, *et al.* compared the effect on food intake and satiety after intraduodenal infusion of fat and CHO between obese and non-obese men [34]. Intraduodenal infusion of fat and CHO suppressed appetite and food intake in both obese and non-obese men. Thus, in this study, the effects of intraduodenal infusion of fat and CHO on food intake and satiety were not affected by BMI. In addition, Maljaars, *et al.* described altered gut peptide secretion and blunted vagal responses to nutrients when obesity develops [62]. Thus, it can be suggested that obesity leads to altered gastrointestinal responses to nutrients. Still, the effect of an increase in BMI on the intestinal brake is not completely known.

Aging results in a physiological decline in food intake. This phenomenon is called “anorexia of aging”. Cook, *et al.* studied the effect of intraduodenal nutrient infusion (fat and CHO) in older and younger subjects [17]. They observed no significant difference in food intake from a buffet meal between older and younger subjects. Increased feedback from intestinal nutrient infusion in the elderly seems not to be responsible for the “anorexia of aging”. Other factors, for example alterations in taste and smell or changes in the gastrointestinal motility and secretion, may play a role.

Up to now, no data have been published with regard to gender differences in intestinal brake effects.

## 8. Conclusions

There is substantial evidence that all regions of the small intestine are able to generate negative feedback signals that influence the functions of the more proximal parts of the GI tract. Activation of the intestinal brake occurs after reaching a threshold to induce the release of mediators that are involved in intestinal brake activation. The inhibitory effect of this negative feedback mechanism differs between the different sites in the small intestine and the exposed area of infusion. The duodenum, jejunum, and ileum and their specific effects on the inhibition of motility, GI peptide release, food intake, and satiety are frequently evaluated in humans. However, several studies have used a caloric infusion rate that surpassed the normal gastric emptying rate, resulting in increased concentrations of undigested nutrients in the more distal parts of the small intestine. It is very likely that this effect is also created by bariatric surgery procedures such as RYGB, where a high amount of nutrients is delivered into the jejunum and causes chronically sustained ileal brake activation.

Clinical studies evaluating the effect of small intestinal infusion of fat on food intake and satiety are numerous; these studies clearly demonstrate a suppression of food intake and increased satiety. Protein and CHO can suppress food intake and satiety to the same extent as equicaloric amounts of fat. It is concluded that a proximal to distal gradient exists with relation to the magnitude of the intestinal brake effect: the more distal the nutrients and their degradation products in the small intestine, the greater the effect on food intake and satiety (Table 2).

Prolonged activation of the ileal brake by means of naso-ileal tubes is not feasible in daily practice and is therefore not an option as treatment for obesity. Alternatives such as efficient delivery of encapsulated nutrients into the ileum should be explored with focus on efficacy and safety. Taken together, the observations described in this review point to the ileal brake as the most potent small intestinal feedback mechanism for controlling food intake and satiety. Access of nutrients to the distal part of the small intestine is necessary in order to achieve the most potent effects on food intake and satiety. Taking all relevant factors into account, the ileal brake can be seen as a most interesting target for food-based approaches in the continuing battle against overweight and obesity.

## Figures and Tables

**Table 1:** Overview of infusion studies in the different sites of the small intestine and the measured effects on food intake and satiety. CHO, delivery of carbohydrates.

Site *Nutrient*	Caloric Load, kcal/min; Total Amount of Calories, kcal; Nutrient Specifications	Effect on Satiety, Appetite, Hunger, Fullness, and Desire to Eat	Energy Intake, kcal (Mean, SEM)	Energy Intake (± %)	Reference
Duodenum					
*Protein*	0.5 kcal/min; 30 kcal; 18.1% hydrolyzed whey protein	No decreased hunger	1191 ± 113 kcal (control 1237 ± 111 kcal) ^¤^	−2.7%	[28]
	0.5 kcal/min; 30 kcal; 18.1% hydrolyzed whey protein	No decreased hunger No increased fullness	1153 ± 151 kcal (control 1270 ± 150 kcal) ^¤^	−9%	[29]
	1.5 kcal/min; 90 kcal; 18.1% hydrolyzed whey protein	No decreased hunger	1077 ± 125 kcal (control 1237 ± 111 kcal) ^¤^	−13%	[28]
	1.5 kcal/min; 90 kcal; 18.1% hydrolyzed whey protein	No decreased hunger No increased fullness	1118 ± 163 kcal (control 1270 ± 150 kcal) ^¤^	−12%	[29]
	3.0 kcal/min; 270 kcal; 18.5% Whey protein isolate	No decreased hunger	1032 ± 83 kcal (control 1241 ± 80 kcal) *	−17%	[30]
	3.0 kcal/min; 180 kcal; 18.1% hydrolyzed whey protein	No decreased hunger No increased fullness	1031 ± 153 kcal (control 1270 ± 150 kcal) *	−19%	[29]
	3.0 kcal/min; 180 kcal/min; 18.1% hydrolyzed whey protein	No decreased hunger	912 ± 120 kcal (control 1237 ± 111 kcal) *	−26.3%	[28]
*Amino acid*	0.075 kcal/min; 6.75 kcal; l-tryptophan	Increased fullness *	1155 ± 109 kcal (control 1215 ± 107 kcal) ^¤^	−4.9%	[31]
	0.15 kcal/min; 13.5 kcal; l-tryptophan	Increased fullness * Increased desire to eat *	996 ± 122 kcal(control 1215 ± 107 kcal) **	−19%	[31]
*CHO*	1 kcal/min; 90 kcal Glucose 5%	Decreased hunger (compared to jejunal infusion) *	1252 ± 97 kcal (compared to mid-jejunal CHO 1413 ± 62 kcal) ^¥^	−11%	[32]
	1 kcal/min; 120 kcal Glucose 25%	No objective	1195 ± 61 kcal(control 1062 ± 118 kcal)	+12.5%	[33]
	2 kcal/min; 240 kcal; Glucose 25%	No objective	1200 ± 87 kcal(control 1062 ± 118 kcal)	+13%	[33]
	2 kcal/min; 180 kcal; Glucose 25%	Suppression desire to eat *	No significant decrease food intake	Kcal energy intake not mentioned	[27]
	2 kcal/min; 180 kcal; Fructose 25%	No suppression desire to eat	Significant decrease food intake *	Kcal energy intake not mentioned	[27]
	2.86 kcal/min; 343.2 kcal; Glucose 25%	Increased fullness **	896.9 ± 94.6 kcal (control 960.2 ± 85.5 kcal) ^¤^	−3.6%	[34]
	2.86 kcal/min; 343.2 kcal; Glucose 25%	Decreased hunger ***	821.0 ± 61.1 kcal (control 964.0 ± 56.1 kcal) **	−15%	[26]
	2.9 kcal/min; 348 kcal; Glucose 25%	Increased fullness *	No significant decrease in energy intake compared to fat	Kcal energy intake not mentioned	[17]
	3.2 kcal/min; 288 kcal; Glucose 20%	Decreased hunger ** Increased fullness **	1136.2 kcal (control 1536.4 kcal) **	−26%	[25]
	3.2 kcal/min; 288 kcal; Glucose 20%	Decreased hunger * Increased fullness * Increased satiety *	907 ± 150 kcal (control 1093 ± 152 kcal) *	−17%	[23]
	4 kcal/min; 480 kcal; Glucose 25%	No objective	939.9 ± 114.6 kcal (control 1061.4 ± 11 [33] 7.5 kcal) ***	−22%	[33]
*Fat*	0.25 kcal/min; 25 kcal; 10% Intralipid	Decreased hunger^*^	1282 ± 44 kcal (control 1289 ± 62 kcal) ^¤^	−0.5%	[35]
	0.9 kcal/min; 54 kcal; 10% high oleic rapeseed oil in water	No significant effect on satiety	No significant decrease energy intake compared to ileal infusion	Kcal energy intake not mentioned	[9]
	1.33 kcal/min; 66.5 kcal; 10% Intralipid	No significant difference in appetite scores	1346 ± 109 kcal (control 1346 ± 90 kcal) ^¤^	0%	[36]
	1.33 kcal/min; 199.5 kcal; 10% Intralipid	No significant difference in appetite scores	1242 ± 84 kcal (control 1346 ± 90 kcal) ^¤^	−7.7%	[36]
	1.5 kcal/min; 75 kcal; 10% Intralipid	Decreased hunger *	1235 ± 71 kcal (control 1289 ± 62 kcal) ^¤^	−4.2%	[35]
	2.0 kcal/min; 189 kcal; 20% Intralipid	No significant effect on satiety	359 ± 51 kcal (control 521 ± 80 kcal) **	−31%	[37]
	2.86 kcal/min; 343.2 kcal; 10% Intralipid	Increased fullness ***	742.6 ± 120.9 kcal (control 960.2 ± 85.5 kcal) ^¤^	−22%	[34]
	2.86 kcal/min; 343.2 kcal; 10% Intralipid	No significant difference in appetite scores	775.4 ± 80.3 kcal (control 964.0 ± 56.1 kcal) **	−20%	[26]
	2.9 kcal/min; 348 kcal; 10% Intralipid	Decreased hunger ** Increased fullness *	Significant decrease in food intake compared to duodenal glucose *	Kcal energy intake not mentioned	[17]
	3.0 kcal/min; 270 kcal; 20% Intralipid	No decrease in hunger	973 ± 103 kcal (control 1241 ± 80 kcal) *	−22%	[30]
	3.0 kcal/min; 270 kcal; 20% Intralipid	Suppression of desire to eat *	1012.7 ± 107.8 kcal (compared to L2: 1263.5 ± 110.1 kcal and L1: 1303.9 ± 78.8 kcal) *	−22%	[38]
	4 kcal/min; 200 kcal; 10% Intralipid	Decreased hunger *	1139 ± 65 kcal (control 1289 ± 62 kcal) *	−12%	[35]
	4 kcal/min; 200 kcal; 10% Intralipid	No significant difference in appetite scores	1358 ± 132 kcal (control 1346 ± 90 kcal) ^¤^	−1%	[36]
	4.9 kcal/min; 367.5 kcal; 50% corn oil emulsion	Increased fullness *	1377 ± 79 kcal (control 2026 ± 194 kcal) *	−32%	[39]
*Fatty acid*	0.2 kcal/min; 18 kcal; Lauric acid (C12)	No significant difference in appetite scores	1341 ± 130 kcal	No significant difference compared to 0.3 or 0.4 kcal/min	[40]
	0.3 kcal/min; 27 kcal Lauric acid (C12)	No significant difference in appetite scores	1312 ± 65 kcal	No significant difference compared to 0.2 kcal/min	[40]
	0.4 kcal/min; 36 kcal; Lauric acid (C12)	No significant difference in appetite scores	1077.2 ± 112.3 kcal (1339.9 ± 130.2 kcal) *	−20% (compared to 0.2 kcal/min)	[40]
	0.4 kcal/min; 24 kcal; Lauric acid (C12)	No significant difference in appetite scores	1134 ± 80 kcal (control 1265 ± 92 kcal) *	−10%	[41]
	0.4 kcal/min; 24 kcal; Oleic acid (C18)	No significant difference in appetite scores	1249 ± 72 kcal (control 1265 ± 92 kcal) ^¤^	−1%	[41]
Jejunum					
*Protein*	No data	No data	No data	No data	
*CHO*	1 kcal/min; 90 kcal; Glucose 5%	Increased hunger and desire to eat (compared to duodenal infusion) *	1413 ± 62 kcal (compared to duodenal infusion 1252 ± 97 kcal) ^¥^	−11% (compared to duodenal infusion)	[32]
*Fat*	4.9 kcal/min; 367.5 kcal; 50% corn oil emulsion	Decreased hunger *	1076 ± 202 kcal (control 2157 ± 196 kcal) **	−50%	[39]
Ileum					
*Protein*	0.19 kcal/min; 17.1 kcal; Casein	No significant difference in appetite scores	528.4 ± 86.1 kcal (control 586.7 ± 70.2 kcal) ^¤^	−9.9%	[42]
	0.57 kcal/min; 51.3 kcal; Casein	Decreased hunger ***	458.0 ± 78.6 kcal (control 586.7 ± 70.2 kcal) ***	−22%	[42]
*CHO*	0.19 kcal/min; 17.1 kcal; Sucrose	No significant difference in appetite scores	491.4 ± 77.5 kcal (control 586.7 ± 70.2 kcal) ^¤^	−21%	[42]
	0.57 kcal/min; 51.3 kcal; Sucrose	No significant difference in appetite scores	399.0 ± 57.0 kcal (control 586.7 ± 70.2 kcal) ***	−32%	[42]
*Fat*	0.57 kcal/min; 51.3 kcal; Safflower oil	No significant difference in appetite scores	464.3 ± 90.7 kcal (control 586.7 ± 70.2 kcal) ***	−21%	[42]
	0.6 kcal/min; 27 kcal; Safflower oil 6%	Increased satiety *	No objective	No objective	[43]
	0.6 kcal/min; 54 kcal; Oil 10%	Decreased hunger *	422 kcal (control 499 kcal) **	−15%	[44]
	0.9 kcal/min; 54 kcal; 10% high oleic rapeseed oil in water	Decreased hunger *	No significant decrease energy intake compared to duodenal infusion	Kcal energy intake not mentioned	[9]
	1.8 kcal/min; 60.75 kcal; Safflower oil 6%	Increased satiety *	No objective	No objective	[43]
	4.9 kcal/min; 367.5 kcal; 50% corn oil emulsion	Increased fullness *	1377 kcal (control 2026 kcal) *	−32%	[39]
	4.93 kcal/min; 370 kcal; 50% corn oil	No significant difference in appetite scores	1313 kcal (control 1883 kcal)	−30%	[8]

* *p* < 0.05; ** *p* < 0.01; *** *p* < 0.001; ^¥^ *p*: 0.05; ^¤^ *p* > 0.05.

**Table 2:** Summary of the effects of the different macronutrients in the different sites in the small intestine on energy intake.

Site Nutrient	Average Decrease Energy Intake in %; Average Caloric Infusion Rate in kcal/min	Energy Intake	Reference
Duodenum			
*Protein*	20.8%; 3.0 kcal/min	↓	[28,29,30]
*CHO*	20%; 2.85 kcal/min	↓	[23,25,26,32,33]
*Fat*	21.5%; 3.69 kcal/min	↓	[26,30,35,37,39]
Jejunum			
*Protein*	*	*	
*CHO*	11%; 1 kcal/min	↓↓ °	[32]
*Fat*	50%; 4.9 kcal/min	↓↓	[39]
Ileum			
*Protein*	22%; 0.57 kcal/min	↓↓↓	[42]
*CHO*	32%; 0.57 kcal/min	↓↓↓	[42]
*Fat*	21.7%; 2.02 kcal/min	↓↓	[39,42,44]

* No data; ° Directly compared jejunal infusion and duodenal infusion (1413 ± 62 kcal *vs.* 1252 ± 97 kcal); ↓: <10% decrease energy intake at a caloric infusion rate of 1 kcal/min; ↓↓: >10% and <20% decrease energy intake at a caloric infusion rate of 1 kcal/min; ↓↓↓: >20% decrease energy intake at a caloric infusion rate of 1 kcal/min.
